# Cheminformatic Characterization of Natural Antimicrobial Products for the Development of New Lead Compounds

**DOI:** 10.3390/molecules26133970

**Published:** 2021-06-29

**Authors:** Samson Olaitan Oselusi, Alan Christoffels, Samuel Ayodele Egieyeh

**Affiliations:** 1 School of Pharmacy, University of the Western Cape, Bellville, Cape Town 7535, South Africa; segieyeh@uwc.ac.za; 2 South African Medical Research Council Bioinformatics Unit, South African National Bioinformatics Institute, University of the Western Cape, Cape Town 7535, South Africa; alan@sanbi.ac.za

**Keywords:** antimicrobial resistance, natural products, cheminformatics, hit prioritization, hit-optimization, drug-likeness

## Abstract

The growing antimicrobial resistance (AMR) of pathogenic organisms to currently prescribed drugs has resulted in the failure to treat various infections caused by these superbugs. Therefore, to keep pace with the increasing drug resistance, there is a pressing need for novel antimicrobial agents, especially from non-conventional sources. Several natural products (NPs) have been shown to display promising in vitro activities against multidrug-resistant pathogens. Still, only a few of these compounds have been studied as prospective drug candidates. This may be due to the expensive and time-consuming process of conducting important studies on these compounds. The present review focuses on applying cheminformatics strategies to characterize, prioritize, and optimize NPs to develop new lead compounds against antimicrobial resistance pathogens. Moreover, case studies where these strategies have been used to identify potential drug candidates, including a few selected open-access tools commonly used for these studies, are briefly outlined.

## 1. Introduction

The advent of antibiotics in the 20th century has been a significant turning point in medical sciences and humanity [1]. Many antibiotics were discovered and developed for human use twenty years after the second world war [2]. This golden era (the 1940s to 1970s) is remembered for the rise of antibiotics in transforming human health by saving many lives through the treatment of infectious diseases [2,3]. However, the few antibiotics developed after the period were derivatives of the existing ones. The situation was compounded by the sudden emergence of antibiotic-resistant pathogens [1,4]. This condition has resulted in a global burden of bacterial infections to a significant threat level, especially among those pathogens, which cannot be controlled using the old classes of antimicrobial agents [5,6]. Therefore, there is a need for the discovery and development of novel antibiotics.

Natural products (NPs) have continued to gain relevance in the battlefront against infectious diseases. Newman and Cragg [7] studied the use of NPs as sources of novel drugs approved between 1981 and 2019. The authors concluded that these compounds have prospects for discovering new agents against various infectious diseases. An earlier study conducted by Seyed [8] also reported the potential of NPs as antimicrobial agents acting against a wide range of human diseases. The efficient exploration of libraries of NPs using modern drug discovery techniques, such as cheminformatic characterization can help identify potential antibiotics.

Several cheminformatic techniques have been developed and employed in drug discovery, design, and development to reduce the research cycle and minimize the cost of producing new anti-infective agents [9]. Generally, the cheminformatics approach to rational drug design involves the estimation of pharmacokinetic and toxic properties of potential drug candidates, with the prospect of minimizing the risk of future attrition [10,11,12]. Here, we reviewed natural products with antimicrobial activities and described the role of cheminformatics characterization in hit profiling, hit prioritization, and hit optimization for antimicrobial development. 

## 2. Natural Products in Antimicrobial Drug Discovery

Compounds sourced from natural products (NPs) have proven to be promising in the discovery and development of novel antimicrobial drugs [13,14]. These compounds are obtained from living organisms, such as bacteria, fungi, plants, and marine microorganisms [15,16]. Studies have reported that four-fifths of the population in most developing nations live on trado-medical practices as the primary source of treatment in essential healthcare services [17,18]. The approval of some NP-based therapies against a range of diseases, such as Alzheimer, cancer, diabetes, and other infections was extensively discussed in another study [19]. Furthermore, three out of the five newly developed drugs by the United States Food and Drug Administration (FDA), representing novel classes of antibiotics between 1981 and 2010, were also sourced from NPs [20]. Therefore, there has been increasing interest in exploring and pursuing NPs as promising lead compounds in combating multidrug-resistant bacteria [18,21]. 

The antimicrobial potential of crude extracts and pure NPs has been studied by observing the growth response of pathogens to samples. Table 1 shows selected NPs with their reported bioactivity against some antimicrobial-resistant bacteria. The selection criteria of promising antimicrobial compounds are based on minimum inhibitory concentration (MIC) values of not more than 100 μg/mL and 25 μM for crude extract, and pure compounds, respectively [22,23,24].

Despite the availability of these bioactivity data for natural products against resistant bacteria, virtually none have been developed into an antimicrobial drug candidate. This might be due to the difficult, broad, risky, costly, and time-intensive process of drug discovery and development [9,38]. Therefore, it has become imperative to embrace the available knowledge to quest for faster, cheaper, and more effective drug discovery and development approaches.

## 3. Cheminformatics Techniques in Antimicrobial Drug Discovery and Development

Drug developers are employing different modern strategies to overcome the challenges. These current drug discovery and design strategies can computationally identify potential liabilities and optimize hit compounds to impact desired drug-like properties prior to expensive synthesis and pre-clinical experiments. In addition, it can computationally process a large set of compounds from virtual combinatorial libraries and high-throughput screening to guide rational decision-making in drug discovery and development. This technique of processing large chemical bioactivity data is called cheminformatics [39]. 

### 3.1. Overview of Cheminformatics

**Figure 1:**
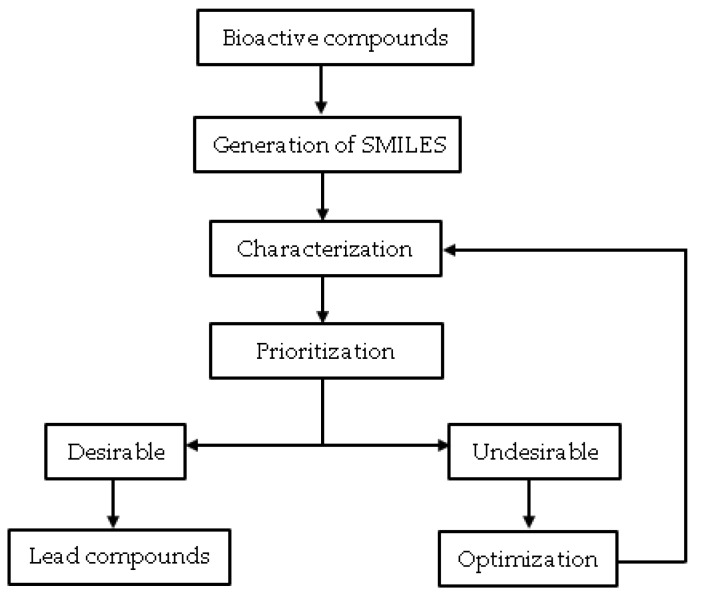
The overall methodology of cheminformatics application in lead discovery.

Cheminformatic is a data mining technique that uses computer and information strategies to solve chemical problems by processing raw data into information and information into knowledge [39,40]. Chemical data processing in this context involves working with chemical structures [41]. Therefore, this strategy for drug developers aims to provide better and faster decision-making processes in discovery and lead optimization [39]. Cheminformatics is gaining much acceptance in the field of computational chemistry. It has great potential, especially in the retrieval and extraction of chemical information, database search for compounds, interactive data mining for molecular graphs, and analyses of chemical diversity [39,41,42,43]. It is relevant, particularly in processing hit compounds from virtual and actual high throughput screenings. Cheminformatic processes such as hit profiling (assessing physiochemical properties, molecular descriptors, and drug-likeness) can guide hit prioritization and hit optimization to identify lead compounds (Figure 1), especially from phenotypic screening.

#### 3.1.1. Hit Profiling: Physicochemical Properties of NPs

Cheminformatics have played a significant role in the identification of NPs that has the potential to become drug candidates [44]. These techniques are widely used to support traditional wet-lab experiments towards the early identification of drug-like hit, hit-to-lead, and lead optimization processes while improving potency and selectivity. For example, various structural and molecular representations in cheminformatics have proven to help study the molecular complexity and quantify the chemical diversity of a library of compounds. This computational approach has also allowed for profiling, prioritization, and comparison of the molecular descriptors, physicochemical, and pharmacokinetic properties of a group of NPs and others or with those of known drugs [44,45,46,47].

The evaluation of the physicochemical parameters (PP) of potential drug candidates is crucial in drug development, as it assists in the early identification of molecules that may fail at a later stage [48]. The absorption or therapeutic action elicited by a drug depends mainly on the interaction between the various physical and chemical properties of the drug and the targets [49]. Therefore, the physical and chemical properties of any compound are crucial to evaluate the drug-likeness. Furthermore, PP can be manipulated to an optimized condition using computer-aided strategies for a better drug-receptor relationship. The PP that is key to determining the biological activity of any drug candidate has been reviewed [48,49,50,51,52,53], a few of these properties are discussed below.

##### Molecular Weight (MW)

Molecular weight (MW) is one of the commonly examined physicochemical properties in drug discovery research [54]. This property has been widely studied for its ability to influence various pharmacokinetic properties like absorption, bioavailability, permeation, and elimination, particularly with respect of compounds that are intended for oral administration [55]. MW and few other properties are used in various rule-based drug-likeness filters, such as Lipinski [56] and Ghose [57] to remove undesired compounds from a library. However, antibacterial agents have been reported to deviate from these rules as marketed antibacterial drugs have higher molecular weights than other drugs [58,59]. Furthermore, most marketed antibacterial agents like streptogramins, macrolides, and daptomycin, commonly used against Gram-positive bacteria, possess larger MW than those used against Gram-negative groups [59,60]. However, few Gram-negative bacteria drugs are characterized by substantially high MW. Polymyxin B1 (1203 Da) and azithromycin (749 Da) are examples of these drugs, and they require penetration enhancers to aid their permeability [59]. 

##### Partition Coefficient (logP)

The partition coefficient (logP) is the ability of an uncharged molecule to dissolve in a nonhomogeneous two-phase system of lipid and water [61]. It measures the amount of solute that mixes in the water against that which dissolves in a lipophilic portion. The logP is used to evaluate how a molecule travels to the target from the site of administration [49,61]. This implies that the values of logP are significant indicators of the fate of an administered drug in the target organism. A negative logP indicates that the molecule is more hydrophilic, and a positive logP shows that the molecule has a higher affinity for the lipophilic phase.

Similarly, zero logP means that the substance is equally partitioned between the bi-phasic system [61,62]. In order to achieve the desired antimicrobial efficacy, it is important to identify or design compounds with optimum logP that will ensure efficient penetration of the microbes’ cell wall by the natural products. High permeability through microbial cell wall increase efficacy while decreased permeability may give rise to antimicrobial resistance. The ideal logP of active molecules against Gram-negative bacteria was around four, and six, respectively [63]. 

##### Hydrogen Bonding

Hydrogen bonding refers to the relationship of an atom of hydrogen from a given compound (known as the donor) and a hydrogen atom from different compounds (known as acceptor), evidenced by bond formation [64,65]. Hydrogen bonds (HBs) are crucial in evaluating the specificity of the binding of a ligand substance to a receptor. The importance of hydrogen bonds in determining the specificity of drug binding has been reported in various studies [66,67,68]. The impact of HBs in the analysis of the quantitative structure-activity relationships (QSAR) model has also been established [49,64]. For example, Kemegne et al. [69] studied the antimicrobial structure-activity relationship of anthraquinones isolated from *Vismia laurentii*. They reported that hydrogen bond acceptors of the compounds were a determinant of their antimicrobial activity. Furthermore, the addition of a properly positioned HBA side chain (to form an intramolecular HB) may be logical when hydrogen bond donors are required for target activity [70]. Hence, quantifying HBs is vital in identifying and optimizing hit compounds [57]. 

### 3.2. Concept of Drug-Likeness

Drug-likeness is a quantitative concept used to describe molecules that possess functional groups, chemical and physicochemical properties consistent with most of the approved drugs [71,72]. It provides an insight into the early identification of chemical compounds that are “most likely to succeed” in the drug development venture. A commonly used approach for estimating the drug-likeness of a given molecule is to screen against acceptable boundaries of some fundamental molecular properties. An example of this strategy is the famous “Rule of Five’’ developed by Lipinski et al. [56]. Ghose [57] and Veber’s rule [73], among many other property-based rules, have also been used in various studies to determine drug-likeness [74]. The question is whether the application of these drug-likeness estimation strategies to natural products is a comparison of apples with oranges? Natural products, chemical entities produced by living organisms, tend to break these established drug-likeness rules obtained from synthetic chemical libraries. The concept of natural product-likeness has been reported to have the potential to open new opportunities for drug discovery from natural compounds while neglected by the drug-likeness rule [72]. 

#### 3.2.1. Lipinski’s Rule of Five (Ro5)

The Ro5 is a collection of some important PP that needs to be prioritized in determining the success of orally administered drugs [49,75,76]. There are a likelihood for poor absorption and permeability for drug candidates whose logP, hydrogen bond donors (HBDs), hydrogen bond acceptors (HBAs), and molecular weight (MW) is above 5, 5, 10, and 500, respectively [74,75,76,77]. The digit 5 in Ro5 indicates the limit of the parameters, multiples of 5 [49]. This strategy aims to use a drug-likeness filter to identify for quickly; removal or optimization of poor pharmacokinetic compounds at an earlier stage of drug discovery [74,76,77]. 

Several authors have explained successful cases where Ro5 has been employed to evaluate the drug-likeness of hundreds and thousands of NPs [76,78,79]. Zhang and Wilkinson [80] also reported that about two-thirds of the FDA-approved drugs are administered orally and passed the Ro5. However, some drawbacks have been identified with the use of Lipinski’s rule. For example, approved drugs, such as atorvastatin, bromocriptine, and everolimus are notable violators of the Ro5 [81,82]. Similarly, Zhang and Wilkinson [80] have reported that 20% of all orally administered drugs failed at least one of the parameters of Lipinski’s rule.

Furthermore, the harsh cut-off that is used in Lipinski’s parameters has failed to distinguish between molecules with similar properties [71,83]. In another words, a compound with a MW of 501 Da is considered to have a considerably lower likelihood of success than one with a MW of 499 Da [84]. These constraints can result in significantly missed opportunities [83,84]. Therefore, the Ro5 alone may not be sufficient to evaluate the drug-likeness prospects of many compounds [74].

#### 3.2.2. Pharmacokinetics and Toxicity Parameters

Pharmacokinetic descriptors such as absorption, distribution, metabolism, and excretion (ADME), and toxicity (T) are commonly used properties for profiling or predicting the fate of many drug candidates after clinical administration [85]. The concept of investigating the ADMET is of interest in early drug discovery given that over 70% of clinical failures have been connected to these properties [86,87]. In addition to potency, a successful drug candidate is expected to have favorable ADMET properties [85,87]. 

The use of in silico methods in determining these parameters has significantly contributed to recent advancements in discovery and development [49]. For instance, ADMET profiling has been used in various studies to identify lead compounds [88,89]. In addition, the assessment of the ADMET properties for potential drug candidates could guide computational chemists towards an effective structure-activity relationship (SAR) based optimization [87,90]. 

### 3.3. Hit-Prioritization Using the Quantitative Estimate of Drug-Likeness

To address the constraints of the rule-based filtering of compounds, Bickerton et al. [71] developed a quantitative estimate of drug-likeness (QED) by combining the desirability of key physicochemical properties (such as molecular weight, polarity, numbers of hydrogen bond acceptors, and donors, lipophilicity, and the number of structural alerts) , which impacts the likelihood of attrition [74,91,92]. The QED is a flexible and continuous metric score whose value ranges between 0 and 1. A score of 1 in this context describes any chemical compound with all its physicochemical properties within the space of an ideal oral drug-like profile, while a score of 0 describes a compound with undesired properties [62,92,93] 

The concept of QED has been used in various studies to prioritize large compound sets and their drug targets. For example, Egieyeh et al. [93] conducted cheminformatic profiling of 1040 NPs with anti-plasmodial activity. They generated a list of compounds that can be prioritized in the development of anti-malarial drugs. Similarly, a collection of more than 100 active compounds against methicillin-resistant *Staphylococcus aureus* (MRSA) was also prioritized for anti-MRSA drug development in a recent study [62]. Kim and Lee [94] also screen chemical compounds obtained from a Chinese medicinal plant. They used the QED concept as one of the approaches to profile 475 active compounds for drug-likeness and oral bioavailability. In all these studies, QED has been described as a more reliable method to estimate drug-likeness than the rule-based approaches [74,92]. 

### 3.4. Hit Optimization after Hit Profiling

The aim of structurally optimized hit compounds is to enhance the development of potential drug candidates. In silico cheminformatic tools can help enhance the physicochemical and pharmacokinetic properties of hit compounds. This is achieved by selectively modifying the structure of such compounds [95,96,97]. In general, this strategy also tends to optimize the compounds toward reducing toxicity, improving ADME properties, and synthetic accessibility while maintaining the desired potency [95,96].

Structural optimization in drug design can be carried out through a combination of different approaches [97]. The simplest of these strategies is the direct chemical modification of functional groups through isosteric replacement, addition, and alteration of the ring systems [98]. This strategy is based on the chemical similarity principle, which states that chemically similar structures will have similar bioactivity. In a recent study [62], random replacement of the functional group was performed on two chemical compounds, α-viniferin and aminoethyl-chitosan, which showed good anti-MRSA activity but a low desirability score. This led to the identification of two compounds with a significantly improved properties and a better desirability score.

Similarly, the removal or addition of a halide to a low-potency inhibitor of factor Xa was performed by Wunberg et al. [99]. The authors obtained a new compound, BAY 59-7939 which had a more improved activity. Another optimization approach is through SAR and subsequent SAR-directed optimization. Here, the chemical and biological information of the chemical compounds generates a SAR for rational optimization of hit compounds. These two approaches describe the case of more than 30% of anti-cancer drugs that are analogues of natural products [97,100]. 

The optimization of a natural hit also uses a molecular design based on the core structures to generate a pharmacophore-oriented molecular design [97]. Examples of this strategy, include eliminating redundant chiral centers and scaffold hopping, commonly used to identify novel hits with intellectual properties. Unlike the first two approaches, the core structures of the original compound may change significantly during the last approach [97].

### 3.5. Cheminformatics Language and Open Access Software Packages for Hit Characterization, Prioritization, and Optimization

The advent of technology in drug discovery has ushered in various computer-readable chemical representations [101]. For example, chemical structures are represented in cheminformatics as linear strings of the Simplified Molecular Input Line System (SMILES). The SMILES is a line notation language widely used to represent the chemical structure effectively read and processed across various computational systems [101,102]. Most cheminformatics software and online platforms are designed to generate or accept SMILES for calculating essential molecular descriptors, drug-likeness, and other related algorithms. The various strategies described in this review can be achieved using software available as open-access, web servers, or commercial packages. The open-access or webserver tools commonly employed in cheminformatics studies are described in Table 2. A comprehensive compilation of the free and commercial software packages, databases and other in silico drug design tools can also be found at click2drug [103] and vls3d [104]. 

## 4. Conclusions

In light of the growing antimicrobial resistance (AMR), it has become imperative for researchers to stay ahead of this impending global pandemic by developing newer and more potent antibiotics. Although many NPs have proven to have the potential of being developed into new antimicrobial drug candidates, the high financial implications, cost in time, and attrition rates, commonly associated with drug discovery and development, are limiting this venture, especially within the academic research places. Computational strategies, such as cheminformatic characterization offer the potential to resurrect many valuable NPs from the graveyard for antimicrobial hit identification and enhance the progress towards hit-to-lead optimization, as well as the eventual development of potent antimicrobial drug candidates. Some of the methods reviewed here have been used to identify new therapeutic interventions against various pathogens, such as the inhibitors of matrix protein (VP40) in Ebola virus [12]. The cheminformatics methods have also played a significant role in pandemic-related studies, including the ongoing COVID-19 research [113]. However, in vitro, or in vivo techniques are crucial in validating cheminformatic hypotheses as this could guide drug developers in receiving less false-positive results.

## Figures and Tables

**Table 1:** Selected natural products with their reported antimicrobial activity.

SN	Natural Compound	Structure	Source of Compounds	Pathogen	Average Reported MIC (μg/mL) Value	No of the Tested Strains	Reference
1	Resveratrol	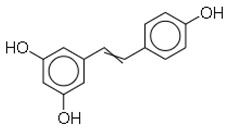	Fruits such as grapes, peanuts, and cranberries	Methicillin-resistant *Staphylococcus aureus* (MRSA)	1.25	3	[25,26]
2	Pterostilbene	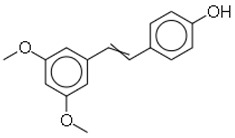	Majorly found in fruits such as blueberries	MRSA	0.078	2	[26,27]
3	7-amino-4-methylcoumarin	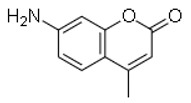	Endophytic Xylaria	*Shigella* *flexneri*	6.3	1	[28]
4	Quercetin	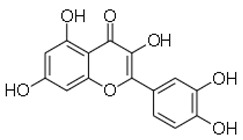	Plants (Guss extracts)	MRSA	31.2–125	29	[29]
5	Anthracimycin	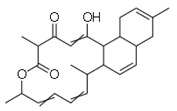	Marine actinobacteria	*Bacillus anthracis*	0.031	1	[30]
6	Protocatechuic acid	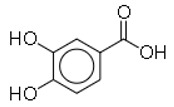	Plants and mushroom	*Escherichia coli*	1	1	[31]
7	Juncuenin D	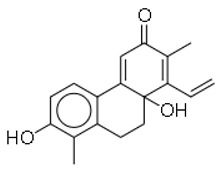	Plants (Juncaceae family)	MRSA	12.5	1	[32]
8	Sanguinarine	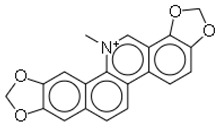	Plants (Papaveraceae families)	MRSA	3.12–6.25	15	[33,34]
9	Vanillic acid	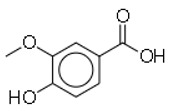	Mushroom	*Escherichia coli*	0.5	1	[31]
10	Abyssomicin C	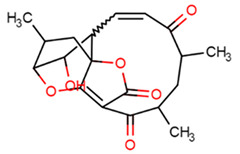	Marine bacterium (Verrucosispora AB-18-032)	MRSA	4	1	[35]
11	MC21-A(Bromophene)	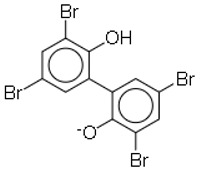	Marine bacterium (*Pseudoalteromonas phenolica*)	MRSA	1–2	10	[35]
12	Canthin-6-one	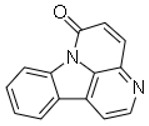	Plant (*Allium neapolitanum*)	*Mycobacterium smegmatis* 14468, *M. phlei* ATCC 11758, *Staphylococcus aureus* 1199B and *S. aureus* XU212	8	4	[28]
13	Psoracorylifol A	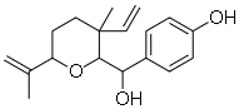	The seeds of *Psoralea* *corylifolia*	*Helicobacter pylori*	12.5–25	2	[28]
14	Erycristagallin	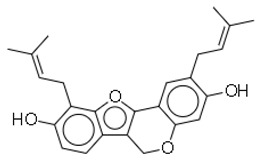	The stem of a plant (*Erythrina subumbrans*)	MRSA	0.78–1.56	4	[28]
15	Hardwickiic acid	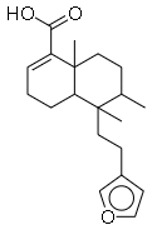	The stem bark of the plant (*Irvingia gabonensis*)	MRSA	19.53	1	[28]
16	Mangostanin	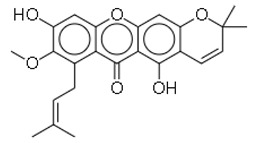	Fruit (*Garcinia cowa*)	MRSA	4.0	1	[28]
17	Mutactimycin C	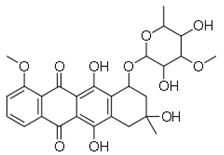	Saccharothrix sp.	*Microccocus leteus* and *Klebsiella pneumoniae*	5	2	[28]
18	Protocatechuic acid	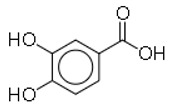	Plants and mushroom	MRSA	1	1	[31]
19	Gancaonin G	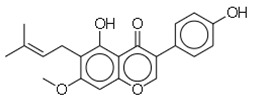	Plants *(Glycyrrhiza uralensis*)	MRSA	16	4	[36]
20	3′-(γ,γ-dimethylallyl)-kievitone	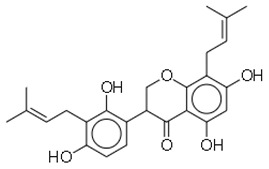	Plants *(Glycyrrh uralensis)*	MRSA	8	4	[36]
21	Licoisoflavone B	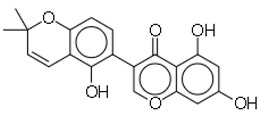	Plants *(Glycyrrhiza uralensis)*	MRSA	32	2	[36]
22	Cryptotanshinon	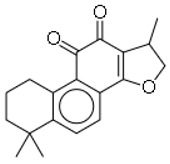	Root of *Salvia miltiorrhiza*	MRSA	0.5–8	16	[37]

**Table 2:** Open access in silico tools for cheminformatics characterization, prioritization, and optimization of hits. All the URL were accessed on the 29 May 2021.

Tool Name	Function	Algorithm	Identifier	Reference
ADMETlab	Drug-likeness evaluation, profiling of ADMET, and subsequent prioritization of chemical entities	Random Forests (RF), Support Vector Machine (SVM), etc.	http://admet.scbdd.com/	[103]
DruLiTo	Physicochemical properties, Drug-likeness rules, QED score	SVM, QSAR	http://www.niper.gov.in/pi_dev_tools/DruLiToWeb/DruLiTo_index.html	[104,105]
Drugmint	Predicting the drug-likeness, QED score, and optimization	SVM	http://crdd.osdd.net/oscadd/drugmint/	[106]
SwissADME	Physicochemical properties, ADME, Rule-based drug-likeness, and Optimization	SVM and Bayesian techniques	http://www.swissadme.ch/	[107]
SwissBioisostere	Optimization	Hussain-Rea algorithm	http://www.swissbioisostere.ch/	[108]
pkCSM	Physicochemical properties, Rule-based drug-likeness, ADMET parameters	Graph-based structural signatures	http://structure.bioc.cam.ac.uk/pkcsm	[109]
DataWarrior	Physicochemical properties, Rule-based drug-likeness, Toxicity prediction, prioritization, and optimization (through the generation of Structure−Activity Landscape Index)	Stereo-enhanced Morgan-algorithm	https://openmolecules.org/datawarrior/	[110]
Galaxy	Physicochemical properties, QED score	Structural similarity	https://usegalaxy.eu/	[71]
BioTransformer	Prediction f drug metabolism	Machine learning algorithms	www.biotransformer.ca	[111]
Knime	Molecular descriptors and ADME	Machine learning	https://www.knime.com/	[112]

## Data Availability

The study did not require any data.
